# Serum Renalase Levels in Adolescents with Primary Hypertension

**DOI:** 10.1007/s00246-018-1891-y

**Published:** 2018-05-10

**Authors:** Marta Lemiesz, Edyta Tenderenda-Banasiuk, Dorota Sosnowska, Katarzyna Taranta-Janusz, Anna Wasilewska

**Affiliations:** 1 Department of Pediatrics and Nephrology, Medical University of Bialystok, 17 Waszyngton Street, 15-274 Białystok, Poland; 2 Department of Obstetrics - Gynecology, Medical Hospital in Garwolin, Garwolin, Poland

**Keywords:** Blood pressure, Children, Renalase, Uric acid

## Abstract

The prevalence of hypertension in pediatric populations continues to rise. Recent studies suggest that renalase plays an important role in blood pressure regulation. The aim of this study was to evaluate serum renalase concentrations in hypertensive children. This study was a prospective cohort analysis of 88 adolescents (40 girls; 48 boys) aged 11–18 years, divided into two groups: HT—38 subjects with primary hypertension; and R (reference group)—50 subjects with normal blood pressure. Serum renalase concentration was measured using a commercial enzyme-linked immunosorbent assay kit. Hypertensive patients had higher serum renalase levels (median 29.8 µg/mL; Q1–Q3: 26.1–35.8) than the reference group (median 26.8; Q1–Q3: 22.96–29.4, *p* < 0.01). Serum renalase was strongly related to serum uric acid levels. In hypertensive patients, serum renalase was positively correlated with 24-h systolic blood pressure (SBP) and 24-h diastolic blood pressure (DBP) and with 24-h SBP and 24-h DBP Z-score (LMS). Our results allow us to conclude that serum renalase correlates with blood pressure elevation. Special attention should be drawn to the correlation between renalase and serum uric acid levels not only in hypertensive, but also in normotensive teenagers. Further studies are needed to answer the question of whether increased serum renalase may be a predisposing factor to hypertension in normotensive patients with hyperuricemia.

## Background

While the burden of hypertension in adults is widely known, it is becoming a growing problem among children and teens. Current estimates describe up to 5% of children as hypertensive [1], and the American Heart Association reports that up to 15% of adolescents have abnormal blood pressure (BP), defined as > 120/80 mmHg [2]. In recent years, renalase, a new flavoprotein, has been shown to be involved in the regulation of blood pressure and cardiovascular function [3]. Renalase is strongly expressed in the kidney, but is also present in the heart, skeletal muscle, liver, adrenals, endothelium, peripheral nerves, central nervous system, and also human adipose tissue [4]. In the kidney, it is mainly produced in the proximal tubule, but it is also found in the glomerulus and distal tubule. The kidney produces the majority of the circulating form, and a very high concentration of the renalase is found in the urine. Circulating prorenalase is quickly activated by high catecholamine levels or by an increase in blood pressure [5, 6]. It is known that renalase metabolizes dopamine most efficiently, followed by epinephrine, and then norepinephrine.

Additionally, it has been shown that renalase may have a significant hemodynamic effect in vivo; for example, it may decrease cardiac contractility and heart rate [6]. Results of experimental studies strongly suggest that renalase deficiency might be related to excess dopamine, epinephrine, and norepinephrine states, and accordingly to elevated blood pressure and hypertension; however, recent clinical studies have not confirmed this observation [7].

These conflicting results signal a need for further investigation. To the best of our knowledge, only a few studies on renalase in pediatric hypertensive populations have been published thus far.

The aim of our study was to examine serum renalase concentration in adolescents with primary hypertension and whether it correlates with blood pressure and serum uric acid.

## Methods

The current prospective cohort study was approved by the ethics committee of the Medical University of Białystok, Poland, in accordance with the Declaration of Helsinki. Informed consent was obtained from parents or guardians of all participants and from children older than 16 years.

The study included 38 hypertensive adolescents (11 female and 27 male) aged 11–18 years, who were referred to our unit (Department of Pediatrics and Nephrology, Medical University of Białystok, Poland) for further diagnostics between June 2012 and December 2013. The reference group (R) consisted of 50 age-matched normotensive, healthy teenagers. Clinical histories and blood samples were collected at the study site.

### Identification of Patients

Patients who met all the following inclusion criteria were enrolled in the study: (1) age 11–18 years; (2) primary arterial hypertension, defined as systolic (SBP) and/or diastolic blood pressure (DBP) ≥ 95th percentile, measured on three or more occasions [1]; (3) no clinical or laboratory signs of infection; (4) normal levels of cortisol, thyroid-stimulating hormone (TSH), and renal function; (5) lack of proteinuria; (6) lack of antibiotic within the prior 4 weeks; and (7) signed informed consent. Patients with a history of heart failure, renal or hepatic dysfunction, diabetes mellitus, systemic inflammatory conditions, autoimmune diseases, clinical or laboratory signs of secondary hypertension (documented thyroid, kidney, or heart disease, abnormal Doppler of the renal arteries), oral contraceptive use, current hypertensive therapy, or on medications known to affect serum uric acid levels and blood pressure values were excluded from the study.

### Identification of the Reference Group

The inclusion criteria for the reference group were as follows: (1) female and male patients aged 11–18 years who were attending the general pediatric nephrology outpatient clinic at the Department of Pediatrics and Nephrology, Medical University of Bialystok, Poland; (2) signed informed consent. Health status was determined by the subjects’ medical history and routine laboratory examinations were performed to rule out the presence of acute or chronic disease.

For each subject, a careful clinical history and physical examination were performed. Body weight and height were measured using a balance beam scale and a pediatric wall-mounted stadiometer, and body mass index (BMI) was calculated. Age- and height-specific reference values for BMI and height were generated by the LMS method, which describes the distribution of a measurement Y by its median (M), the coefficient of variation (S), and a measure of skewness (L) required to transform the data to normality [2, 8]. The LMS values were taken from the OLAF study, published by Kulaga et al. [9]. Ambulatory blood pressure monitoring (ABPM) was performed using the oscillometric boso TM-2430 PC2 device (Bosch + Sohn GmbH, Jungingen, Germany). The monitors were programmed to measure BP every 15 min during waking hours (8:00–22:00) and every 30 min during sleeping hours (22:00–8.00). The periods were corrected according to the patients’ diaries. Readings with a minimum 80% of measurement and without a break longer than 2 h were considered sufficient. The mean SBP and DBP were calculated separately for the 24-h and for awake and asleep periods. Additionally, systolic blood pressure load (SBPL) and diastolic blood pressure load (DBPL) during the day and night were calculated. Hypertension (HT) was defined as a mean SBP level ≥ 95th percentile (1, 5 SDS) and SBPL or DBPL load  > 25% [8, 10].

Office blood pressure measurements were taken using an automated oscillometric device (Datascope Accutorr Plus; Mindray DS USA, Inc., Mahwah, NJ, USA) that had been validated for use in children. Four cuff sizes were available (child, small adult, adult, and large adult). The appropriate cuff size (bladder width at least 40% of arm circumference and length 80–100% of arm circumference) was determined by measuring the mid-upper arm circumference. BP was measured three times at 3-min intervals after a 5–10-min rest in the sitting position with the arm and back supported. The mean of the second and third measurements was used for analysis. All measurements were taken by trained staff.

After overnight fasting, 5 mL of venous peripheral blood was collected from each patient and healthy controls for routine laboratory testing. Isolated serum aliquots were stored at − 80 °C for further analysis. The biochemical work-up included serum creatinine, urea, serum fasting glucose, lipid profile, and serum uric acid concentration. Patients with a serum (UA) > 5.5 mg/dL were considered hyperuricemic [11].

The serum renalase concentration was measured using a commercially available FAD-dependent amine oxidase (RNLS) enzyme-linked immunosorbent assay (ELISA) kit (USCN Life Science Inc., China), according to the manufacturer’s instructions. Serum renalase levels were expressed in µg/mL.

Serum creatinine was determined by the Jaffé reaction, and uric acid was measured using a Hitachi apparatus (Hitachi, Tokyo, Japan). The estimated glomerular filtration rate (eGFR) was calculated using the Schwartz formula: eGFR = *k* × *H* (cm)/*Scr* (mg/dL), where *k* is the age-dependent coefficient (0.55 in boys aged < 12 years and girls of any age, 0.70 in boys aged > 12 years), *H* is height, and *Scr* is the serum creatinine level. Serum cholesterol, HDL cholesterol, and triglycerides were determined by an enzymatic method using a Roche Hitachi 912 analyzer (Roche Diagnostics, Rotkreuz, Switzerland). Serum glucose was measured with the COBAS Integra 800 analyzer (Roche). The 24-h urinary albumin excretion rate (UAER) was analyzed by radioimmunoassay (RIA).

The Shapiro–Wilk test was used to determine normal distribution. Normally distributed data were presented as mean and standard deviation, while non-normally distributed variables were expressed as median and quartile. Comparisons between two groups were performed using Pearson's chi-squared test for categorical variables and the Student *t* test or Mann–Whitney *U* test for continuous variables. Correlations between variables were evaluated by Spearman’s test as appropriate. A *p* value < 0.05 was considered significant. Multivariate logistic regression analyses were performed to detect relationships between categorical data with evaluation of the regression beta coefficients.

Statistica version 10.0 software (StatSoft Inc., Tulsa, OK, USA) was used for statistical analyses.

## Results

The clinical characteristics of HT patients and healthy controls are summarized in Table 1. The age of the teenagers did not differ significantly between groups (*p* > 0.05). There were more boys than girls in both the HT and reference groups (M: 22; F: 16 and M: 26; F: 24, respectively; *p* > 0.05). The BMI Z-scores of children from the HT group were higher than those in the reference group (*p* < 0.01).

**Table 1:** Clinical characteristics of HT patients and healthy controls (R)

Variables	Median (Q1–Q3)		*p*
	Patients (HT)	Reference group (R)	
Age (years)	16.3 (14–17)	15 (12–17)	NS
BMI (Z–score)	1.3 (0.6–1.9)	0.5 (–0.06 to 1.6)	< 0.01
Office SBP (mmHg)	135 (130–139)	123 (114–131)	< 0.01
Office DBP (mmHg)	78 (74–85)	69 (62–75)	< 0.01
Serum uric acid (UA) (mg/dL)	5.9 (5.2–6.8)	4.7 (3.75–5.7)	< 0.01
Renalase µg/mL	29.8 (26.1–35.8)	26.8 (22.96–29.4)	< 0.01
Fasting plasma glucose (FPG) (mg/dL)	90 (85–95)	90.5 (83–94)	NS
Triglycerides (TG) (mg/dL)	100 (66–137)	80 (67–88)	NS
Total cholesterol (mg/dL)	172 (145–194)	162 (142–189)	NS
HDL (mg/dL)	52 (43–62)	46 (40–64)	NS
Albuminuria (mg/24 h)	5.75 (1.93–8.74)	11.7 (7.45–128)	NS
SBP (mmHg)			
Daytime	139 (131–144)	127 (118–132)	< 0.01
Nighttime	124 (119–128)	111 (106–118)	< 0.01
DBP (mmHg)			
Daytime	75 (69–78)	70 (67–73)	< 0.01
Nighttime	62.5 (59–66)	59 (54–63)	< 0.05
SBP load (%)			
Daytime	69.3 (52–82.8)	38 (14.3–52.8)	< 0.01
SBP 24-h Z-score LMS	2.2 (2–2.8)	1.1 (0.7–1.4)	< 0.01
DBP 24-h Z-score LMS	0.9 (0–1.4)	0.3 (− 0.4 to 0.7)	< 0.01

*BMI* body mass index, *Office SBP* systolic blood pressure, average calculated from three independent measurements, *Office DBP* diastolic pressure, average calculated from three independent measurements, *Load* percentage of BP values that exceed the paediatric ambulatory 95th percentile, *NS* not significant

Values are presented as the median with the interquartile range (Q_1_–Q_3_)

**Fig. 1:**
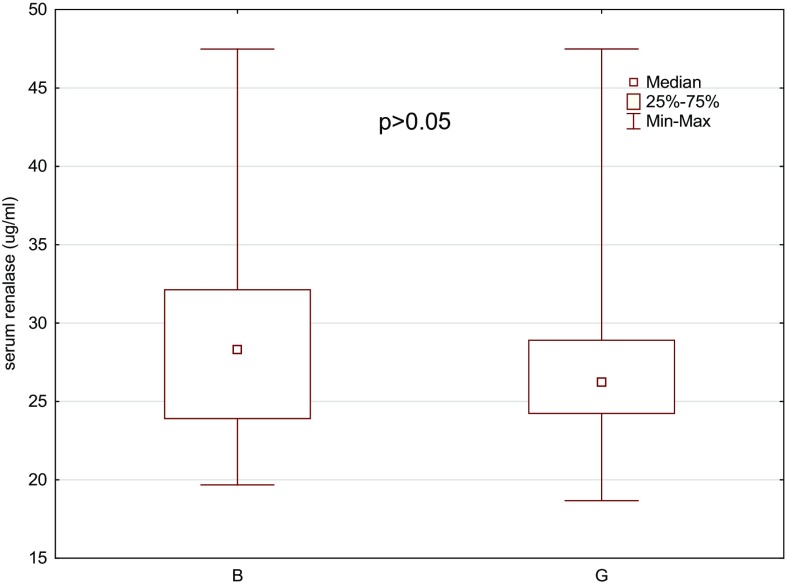
Comparison of renalase serum level between boys (B) and girls (G) (*p* > 0.05)

Compared to the values in the group of healthy controls, office SBP and DBP, 24-h SBP and DBP, Z-score, awake and asleep SBP and DBP, and awake and asleep SBP load were significantly higher in the HT group. There were no significant differences in awake and asleep DBP load or systolic and diastolic nocturnal dip between HT patients and healthy controls. Serum uric acid and renalase levels were significantly higher in hypertensive subjects when compared to the reference group (*p* < 0.01). No statistically significant differences were found in serum renalase between boys and girls (*p* > 0.05; Fig. 1).

**Fig. 2:**
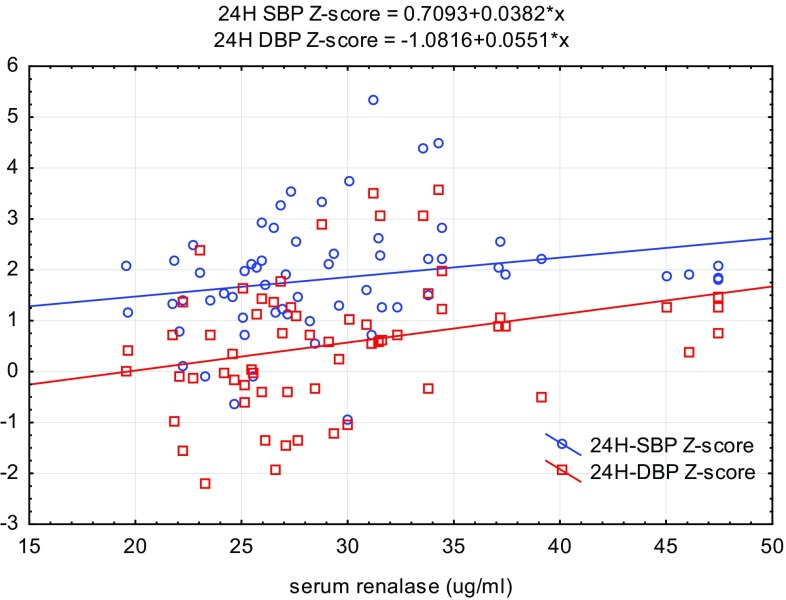
Exponential regression analysis demonstrating the relationship between the renalase concentration and 24-h SBP and 24-h DBP Z-scores in the hypertensive group

**Fig. 3:**
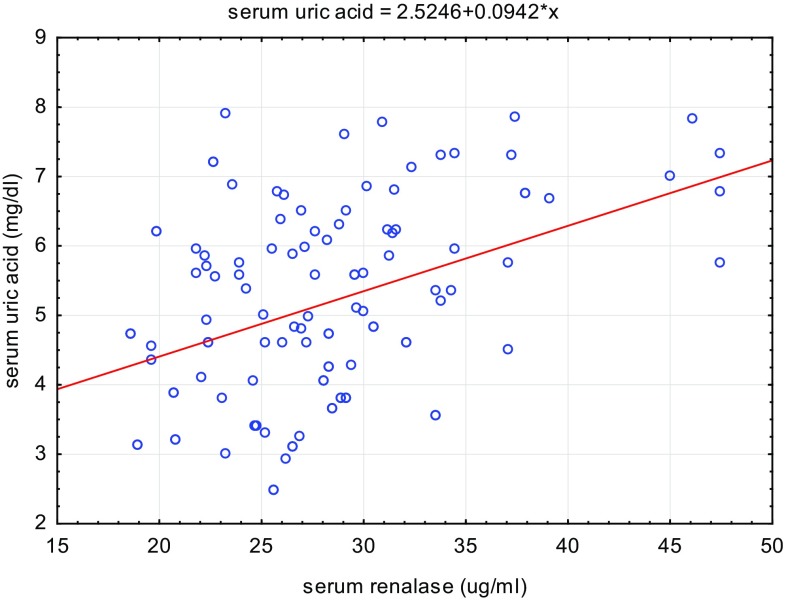
Correlation between the renalase concentration and uric acid (*p* < 0.01)

Univariate analysis of the data revealed positive correlations between serum renalase levels and body weight (*r* = 0.26, *p* < 0.01), BMI Z-score (*r* = 0.2, *p* < 0.05), and serum uric acid level (*r* = 0.37, *p* < 0.01; Table 2 and Figs. 2, 3). We also found statistically significant positive correlations between serum renalase concentration and office SBP (*r* = 0.23, *p* < 0.05) and both 24-h SBP and 24-h DBP (*r* = 0.4, *p* < 0.01; *r* = 0.36, *p* < 0.05, respectively). Additionally, serum renalase correlated positively with awake SBP and DBP (*r* = 0.41, *p* < 0.01; *r* = 0.34, *p* < 0.01, respectively) and asleep SBP and DBP (*r* = 0.32, *p* < 0.05; *r* = 0.27, *p* < 0.05, respectively). The correlations with awake SBPL (*r* = 0.38, *p* < 0.01) and awake DBPL (*r* = 0.37, *p* < 0.01) and asleep DBPL (*r* = 0.28, *p* < 0.05) were also statistically significant. In hypertensive patients, serum renalase was related to 24-h SBP Z-score and 24-h DBP Z-score (*r* = 0.32, *p* < 0.05; *r* = 0.35, *p* < 0.01, respectively) and only awake SBP Z-score (*r* = 0.26, *p* < 0.05).

**Table 2:** Statistically significant correlation between serum renalase and anthropometric values, blood pressure and biochemical parameters

	Correlation coefficient (*r*)	Significance level (*p*)
Weight (kg)	0.26	< 0.01
BMI Z-score (LMS)	0.20	< 0.05
Serum uric acid (mg/dL)	0.37	< 0.01
Office SBP (mmHg)	0.23	< 0.05
Office DBP (mmHg)	0.1	NS
SBP daytime (mmHg)	0.41	< 0.01
SBP nighttime (mmHg)	0.32	< 0.05
DBP daytime (mmHg)	0.34	< 0.01
DBP nighttime (mmHg)	0.27	< 0.05
SBP load daytime (%)	0.38	< 0.01
DBP load daytime (%)	0.37	< 0.01
DBP load nighttime (%)	0.28	< 0.05
24-h SBP	0.40	< 0.01
24-h DBP	0.36	< 0.05
SBP Z-score daytime (%)	0.26	< 0.05
24-h SBP Z-score (LMS)	0.32	< 0.05
24-h DBP Z-score (LMS)	0.35	< 0.01

*Office SBP* systolic blood pressure, average calculated from three independent measurements, *Office DBP* diastolic pressure, average calculated from three independent measurements, *NS* not significant

Serum renalase levels were positively correlated with serum uric acid. To further investigate the correlation between serum renalase and uric acid, patients were divided into two groups according to their serum uric acid level.

**Fig. 4:**
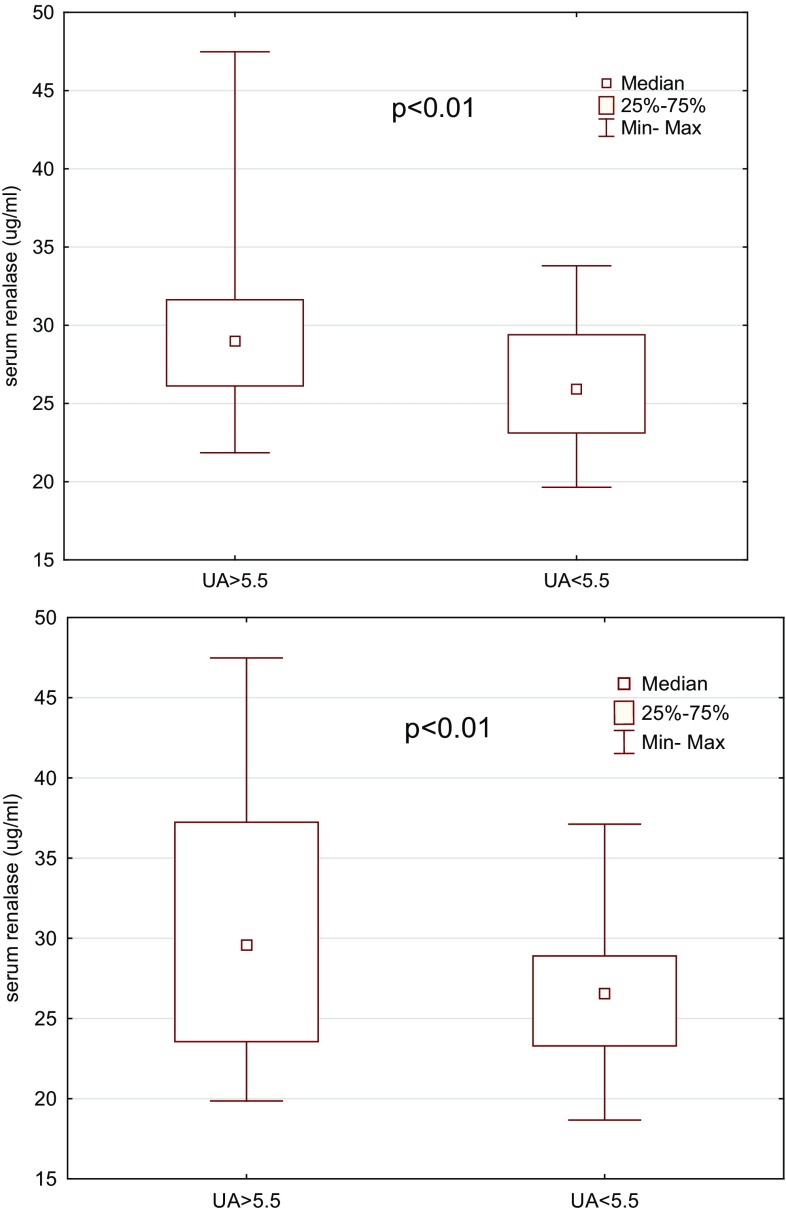
Comparison of renalase serum levels between the patients with hyperuricemia (UA > 5.5) and those without (UA < 5.5) in the hypertensive and reference groups, respectively (*p* < 0.01)

Serum renalase levels were significantly higher in patients with hyperuricemia than those with normal uric acid levels in both the HT and reference groups (Fig. 4).

Factors that were found to correlate significantly with serum renalase concentration in the single regression analyses were used as explanatory variables to create the multiple regression models. In this model, three parameters [serum uric acid, BMI Z-score (LMS), and 24-h SBP Z-score] accounted for more than 25.28% of the variations in renalase levels (*R* = 0.5, *p* < 0.001). As the study group and control group were not matched according to BMI, we performed two additional multiple regression models, which showed that in the model with 24-h SBP Z-score and serum uric acid, the beta coefficient for uric acid was 0.455 and *p* = 0.0001, and in the second model with 24-h SBP Z-score and BMI Z-score, the beta coefficient for BMI Z-score was − 0.04 and *p* = 0.239.

## Discussion

This study demonstrates that serum renalase is significantly higher in hypertensive teenagers when compared with healthy controls. In addition, serum renalase correlates with serum uric acid and is significantly higher in patients with elevated serum uric acid, independent of blood pressure. The etiology of this is not clear.

The prevalence of hyperuricemia is increasing not only in adults but in teenagers as well [12], and is associated with the pathogenesis of hypertension and metabolic syndrome. However, considering the fact that serum renalase was higher in patients with versus without hyperuricemia in both the hypertensive and normotensive groups, we suggest that the relationship between renalase and uric acid may be of clinical significance.

Renalase is a novel amine oxidase discovered in 2005 which is involved in the pathogenesis of hypertension [13]. It is secreted in the blood and is regulated by three key factors: renal function, renal perfusion, and catecholamine levels (dopamine, epinephrine, and norepinephrine) [14]. The relationship between serum renalase and blood pressure has been confirmed in both experimental and clinical studies; however, the results are still equivocal. In a study by Zhao et al. in 2586 Chinese adult individuals, the authors found that two single-nucleotide polymorphisms (SNPs; rs2576178 GG genotype and rs2296545 CC) within the renalase gene were associated with essential hypertension (defined as BP ≥ 160/100 mmHg) [15]. Desir et al. [14] reported that intravenous renalase injection significantly reduced BP in mice, without affecting the heart rate. Xu et al. [3] showed that in rats injected with exogenous recombinant renalase, a mild decrease in SBP, DBP, and mean arterial pressure occurred. The results of the above-mentioned studies indicate that the action of renalase may be attributed to the regulation of the intrarenal dopaminergic system [6]. Renalase was found to play an important role in hypertension associated with kidney disease. In rats subjected to subtotal nephrectomy (5/6 Nx), a single dose of recombinant renalase administered subcutaneously significantly reduced both SBP and DBP [14]. In teenagers, the main determinants of hypertension are visceral obesity and metabolic abnormalities, and it is well known that excessive activation of the sympathetic nervous system is related to visceral obesity. Another major cause is high salt intake. Salt has been shown to modulate the circulating levels of catecholamine, a sympathetic transmitter involved in the regulation of BP. The most important catecholamine in human plasma is dopamine. Dopamine plays an important role in the peripheral sympathetic nervous system, modulates the cardiovascular and endocrine functions, and regulates renal sodium transport, leading to increased blood pressure. Recent studies have shown that renalase can degrade catecholamines and reduce blood pressure. Researchers have also found that sodium may regulate the expression and secretion of renalase, thereby affecting sympathetic nerve activity to modulate blood pressure [3, 16]. Recent experimental study seems to confirm previous observations. Zheng et al. suggested that salt- or potassium-induced changes in blood pressure might be mediated through renalase [17].

In a future study we plan to measure the urine sodium excretion in our patients and investigate correlations with serum renalase levels. The present study was designed to analyze serum renalase levels in relation to blood pressure parameters obtained during 24-h monitoring.

Renalase was presumed to play a role in the pathogenesis of hypertension in experimental models. The relationship between renalase and hypertension was first demonstrated by Zhao et al. [15] in a Chinese population; however, these data were not confirmed in whites [18]. Ficek et al. [4] later showed renalase deficiency in chronic kidney disease and hypertension. Independently, Wybraniec et al. [19] claimed that renalase insufficiency was associated with the presence of hypertension both in patients after surgical repair of aortic coarctation and in a control group.

In this study, we found that serum renalase was significantly higher in hypertensive adolescents than in healthy teenagers and was related to 24-h SBP and DBP, 24-h SBP and DBP Z-score, and daytime SBP load. To the best of our knowledge, this is the first study to investigate the relation between serum renalase and 24-h blood pressure measurement in children and adolescents.

In a study conducted by Maciorkowska et al. [7] in adult patients, the median serum renalase was statistically significantly higher in patients with SBP and DBP > 140/90 mmHg compared to those with readings of < 140/90 mmHg, measured by ambulatory blood pressure monitoring (*p* = 0.018).

Another important finding was a positive relationship between serum renalase and uric acid levels and the BMI Z-score. Uric acid plays an important role in blood pressure regulation. It stimulates both the local [20] and systemic [21] renin–angiotensin system (RAS) and inhibits the release of nitric oxide by endothelial cells [22, 23]. Angiotensin can contribute to increased sympathetic nerve activity, which ultimately results in increased blood pressures [24]. Although the reason for this is not clear, it seems possible that an elevated serum uric acid level, which is quite often observed in children and adolescents with primary hypertension [25], may induce the expression of renalase through the elevated blood pressure levels. Another, more probable mechanism is minor sympathetic and RAS stimulation mediated by oxidative stress, which was documented by Palanisamy et al. [26] in fructose-fed rats. A single oral dose of fructose induced some features of metabolic syndrome in rats.

The catecholamine catabolism induced by renalase directly decreases blood pressure, but also decreases renin secretion and antagonizes the effects of AT1. It has been documented that renalase, which is mainly controlled by the kidney, is a hypotensive agent and fully counterbalances the vasoconstrictive properties of the RAS. The results of recent studies suggest that renalase acts in subtle equilibrium with other systems influencing blood pressure, especially the RAS, and different natriuretic factors.

In summary, our results allow us to conclude that serum renalase may be associated with elevated blood pressure. Special attention should be drawn to the correlation between renalase and serum uric acid levels not only in hypertensive but also in normotensive teenagers.

Our study’s limitations include its single-center, pilot design and the small group of only 38 patients. Therefore, further studies are needed to answer the question of whether increased serum renalase may be a marker indicating a predisposition to hypertension in normotensive patients with hyperuricemia. The ultimate goal is the design of a prospective clinical trial where the predictive powers of renalase levels are used.

## Abbreviations

ABPM: Ambulatory blood pressure monitoring
AT1: Angiotensin receptor
BMI: Body mass index
BP: Blood pressure
CA: Catecholamine
DBP: Diastolic blood pressure
DBPL: Diastolic blood pressure load
eGFR: Estimated glomerular filtration rate
HT: Hypertension
R: Reference group
RAS: Renin–angiotensin system
RIA: Radioimmunoassay
SBP: Systolic blood pressure
SBPL: Systolic blood pressure load
UAER: Urinary albumin excretion rate
